# The dementia severity rating scale: A potential community screening tool for dementia in low- and middle-income countries

**DOI:** 10.1177/14713012231186837

**Published:** 2023-12-14

**Authors:** Yuda Turana, Nicolas Farina, Imelda Theresia, Fasihah Irfani Fitri, Ika Suswanti, Roxanne Jacobs, Marguerite Schneider, Tara Puspitarini Sani, Adelina Comas-Herrera, Emiliano Albanese, Ishtar Govia, Cleusa P Ferri, Martin Knapp, Sube Banerjee

**Affiliations:** School of Medicine and Health Sciences, Atma Jaya Hospital, 64732 Atma Jaya Catholic University of Indonesia, Jakarta, Indonesia; Centre for Dementia Studies, 12190 Brighton and Sussex Medical School, Brighton, UK; Faculty of Health, 6633 University of Plymouth, Plymouth, UK; Alzheimer Indonesia, Jakarta, Indonesia; Department of Neurology, Adam Malik General Hospital, 106100 Universitas Sumatera Utara, Medan Indonesia, Indonesia; School of Medicine and Health Sciences, Atma Jaya Hospital, 64732 Atma Jaya Catholic University of Indonesia, Jakarta, Indonesia; Alan J. Flisher Centre for Public Mental Health, 37716 University of Cape Town, Cape Town, South Africa; 37716 University of Cape Town, Cape Town, South Africa; Alzheimer’s Indonesia, Jakarta, Indonesia; 4905 London School of Economics and Political Science, London, UK; 27216 Università della Svizzera Italiana, Lugano, Switzerland; Caribbean Institute for Health Research (CAIHR)—Epidemiology Research Unit, 54657 The University of the West Indies, Kingston, Jamaica; Department of Psychiatry, 28105 Universidade Federal de São Paulo, Sao Paulo, Brazil; 4905 London School of Economics and Political Science, London, UK; Centre for Dementia Studies, 12190 Brighton and Sussex Medical School, Brighton, UK; Faculty of Health, 6633 University of Plymouth, Plymouth, UK

**Keywords:** dementia, dementia severity rating scale, older adults, screening tools

## Abstract

**Background:**

The Dementia Severity Rating Scale (DSRS) is an informant report, dementia staging tool that is quick to administer and has previous been shown to differentiate between people with dementia and healthy controls. However, it is not clear how accurate the tool is screening against diagnostic criteria in middle-income settings.

**Methods:**

Embedded within the STRiDE programme, older adults (aged ≥65 years) and their informants were randomly recruited from four sites across Indonesia and South Africa. All informants were asked to complete DSRS. We report the tool’s psychometric properties and accuracy against the 10/66 short diagnostic algorithm.

**Results:**

Between September and December 2021, data was collected from 2110 older adults in Indonesia and 408 in South Africa. Overall, the DSRS scores significantly differed between those with and without dementia, as identified on the 10/66 short algorithm (*p* < .05). The difference between groups remained significant after controlling for key factors related to older adult and informant demographics. A score >2 on the DSRS had the greatest agreement with the 10/66 short algorithm and had excellent discriminative properties in both Indonesia (Area Under Curve (AUC) = .75, 95% CIs = .72–.77) and South Africa (AUC = .82, 95% CIs = .76–.88).

**Conclusions:**

The DSRS has potential as a screening tool for dementia in middle-income countries, with high sensitivity and specificity against a standardized diagnostic algorithm.

## Background

The global challenge of rising numbers of people with dementia is well recognized. In large part, this is due to rapid population ageing in low-income and middle-income countries (LMICs) such as Indonesia and South Africa. Worldwide, approximately 50 million people were living with dementia in 2015, which is projected to increase to 152.8 million by 2050 (Nichols et al., 2022), two-thirds of whom will live in LMICs (Prince et al., 2013). Health system and service response is needed to tackle the significant health and social consequences of the global dementia challenge (Prince et al., 2013; 2015). Central to this is a timely and accurate diagnosis, which informs and allows the identification of treatable and reversible forms of dementia, secondary prevention through reduced risk profiles, better management of both cognitive and behavioural and psychological symptoms of dementia, and planning for the future care needs and arrangements (Gauthier et al., 2021).

Brief neuropsychological assessments of cognition, including the Montreal Cognitive Assessment (MoCA) and the Mini-Mental State Examination (MMSE), have demonstrated validity and clinical utility to varying degrees in detecting dementia (Creavin et al., 2016; Larner, 2012; Perroco et al., 2009). However, using cognitive measures alone can be problematic. Many cognitive tasks capture a cross-sectional snapshot of cognitive impairment, rather than the deterioration of cognition necessary in dementia (Taylor-Rowan et al., 2021). These cognitive measures can also be time-consuming, not education-fair, not culturally-fair, and may need well-trained interviewers to ensure standardized and reliable outcomes (Baldo et al., 2013; Li et al., 2012; Nasreddine et al., 2004; Turana & Handajani, 2011). Measures such as the MMSE and MoCA demonstrate good screening properties for Alzheimer’s disease (AD), however, language, educational attainment, literacy, and country of study introduces significant sources of heterogeneity and error (Wang et al., 2022).

The need for education-fair screening tools is particularly pertinent in LMICs such as Indonesia and South Africa, where 13.3% (aged > 50) (BPS-Statistics Indonesia, 2019) and 26.1% (aged 60–64) (Khuluvhe, 2021) are illiterate. Informant-based screening tools therefore have an important role in LMICs due to these measures being less culture and education dependent compared to cognitive tests (Jorm, 2003). Within the dementia literature there are informant-based tools that capture impairment associated with dementia, though their scope and validity, especially cross-culturally, is variable. A recent systematic review of the literature identified that the eight-item interview to ascertain dementia (AD8) and the Informant Questionnaire on Cognitive Decline in the Elderly (IQCODE) have the best screening properties against clinical diagnostic criteria (Taylor-Rowan et al., 2021). Using such instruments requires vigilance about other sources of bias that might be introduced by adopting an informant report. For example, the informant’s relationship to the participant, and informant demographic factors such as culture and ethnicity can influence reports of functional impairment in people with mild cognitive impairment (Burns et al., 2006; Hackett et al., 2020).

The Dementia Severity Rating Scale (DSRS) is a 12-item, informant-report questionnaire assessing various functional and cognitive abilities (Clark & Ewbank, 1996). The DSRS has been widely used across a number of contexts, and benefits from being relatively short (approximately 5–10 min) and cost free to use. The DSRS has been evaluated as a valid means to differentiate cases of AD from health controls (Roalf et al., 2013), and so has the potential to be used as a screening tool, and the informant based DSRS was reported to have a better classificatory accuracy than the MMSE and MoCA. Using the DSRS alongside these measures of cognition can improve diagnostic accuracy (Roalf et al., 2013). Although promising, the performance of DSRS in screening for dementia has not been explored in LMICs, particularly with consideration of other potential sources of bias.

As part of the analysis of data from the STRiDE cohort (Farina et al., 2022) we aimed to ascertain the accuracy of the informant-report DSRS to screen for dementia against a validated diagnostic algorithm in two middle-income countries Indonesia and South Africa.

## Materials and methods

### Participants and setting

This study is part of the wider Strengthening Responses to Dementia in Developing Countries (STRiDE) programme (STRiDE Project, 2018). Data were collected between September and December 2021 in Indonesia and South Africa. Four recruitment sites were chosen: Jakarta, North Sumatra (Indonesia), Cape Town, and Limpopo (South Africa). Sites were selected for pragmatic reasons and to ensure heterogeneity in terms of socioeconomic status and rurality. Random sampling was used across all sites: simple randomisation in the Limpopo site and proportionate to population size (PPS) randomisation in other sites.

Inclusion criteria: age ≥65 years, ability to communicate in one of the target languages (Bahasa, Afrikaans, isiXhosa, Sepedi or English), and availability of an informant (someone who knew the older adult well). There were no criteria related to cognitive impairment or diagnosis of dementia.

Further details about the sampling methodology can be read elsewhere (Farina et al., 2023).

### Measures

Dementia diagnosis (the reference standard) - The 10/66 short schedule (Stewart et al., 2016), upon which the 10/66 short diagnostic algorithm is applied to identify cases of probable dementia. The algorithm has good sensitivity and specificity across a wide range of settings (Abdin et al., 2017; Ibnidris et al., 2021; Khan & Prince, 2022; Stewart et al., 2016). The 10/66 short schedule is composed of the Community Screening Instrument for Dementia (CSI-D) instrument (Hall et al., 2000), the Consortium to Establish a Registry for Alzheimer’s Disease (CERAD) 10-word list learning task with delayed recall (Morris et al., 1989), and the EURO-D, a short, widely used, and validated of depressive symptoms (Prince et al., 1999). The Lawton Instrument of Activities of Daily Living scale (IADL) was used to assess the functional status of the elderly (Lawton & Brody, 1969).

DSRS (the index test) – consists of 12 questions on memory, speaking and language skills, recognizing family members, time orientation, place orientation, decision-making skills, social and community activities. Home activities and responsibilities, personal care and hygiene, eating, continence, ability to move places. The DSRS was administered by a researcher and was not self-completed. In each question section, there are additional questions addressed to the interviewer to rated that they were confident in data collected.

In Indonesia all instruments were presented in Bahasa Indonesia, whilst in South Africa instruments were either Afrikaans, isiXhosa, Sepedi, and English. Details of the cross-cultural adaptation process, alongside other measures collected, are described elsewhere (Farina et al., 2022).

### Procedure

A pair of researchers interviewed the older adult and the informant in the community, typically in the participants' home. Informed consent was obtained for all participants. When participants could not consent, a personal consultee was used to ascertain the older adult’s wishes. A personal consultee is someone the older adult knows and trusts about important life decisions. One researcher interviewed the older adult, whilst the other interviewed the informant. Researchers were trained to interview participants in a manner that allowed for privacy (e.g., interviews completed in separate rooms), however, this was not always possible due to the household environment. In these instances, research would find creative ways to find privacy (e.g., by finding a quiet space outside to complete the interviews). All data were entered into a REDCap database via the REDCap Mobile app (Harris et al., 2009; 2020) on a tablet computer. Ethical clearance was obtained from the London School of Economics and Political Sciences (000834b), the Medical Ethics Committee of Atma Jaya Catholic University of Indonesia (01/12/KEP-FKIKUAJ/2020), the Faculty of Medicine Universitas Sumatra Utara (862/KEP/USU/2020), and the University of Cape Town’s Faculty of Health Sciences Human Research Ethics Committee (HREC 021/2019).

The researchers were blind to the diagnostic algorithm’s outcome as well as the DSRS screening threshold applied, and therefore could not distinguish cases of dementia based on the interviews alone.

### Operational definition

Dementia is characterized by a decrease in at least two domains of cognitive function or behaviour (neuropsychiatric) that interferes with functional activities and is not explained by a significant psychiatric disorder or delirium, with information obtained from the patient’s history or knowledgeable informants with an objective cognitive assessment (McKhanna et al., 2011). In STRiDE, we used the 10/66 short algorithm to identify possible cases of dementia in the community, which was used as a reference standard of community-level dementia diagnosis to validate the index text based on the DSRS assessment.

### Statistical analysis

Only cases that had sufficient data to run the 10/66 short algorithm (i.e., all components of the 10/66 short schedule were completed) were included in the analysis. For example, the algorithm could not be run if the participant declined to complete the EURO-D measure. There is no standardized approach to missing data for the DSRS. All items had negligible missing data (<1%), however, we confirmed that data were missing completely at random (MCAR) through a Little’s MCAR test (Indonesia, *p* = .99; South Africa, *p* = .88). For cases in which there were fewer than 50% of items missing items, the variable mode was imputed in Indonesia (k = 53) and South Africa (k = 13).

Descriptive statistics were used for demographics related to this study (i.e., age, sex, education attainment, cohabiting informant, familial informant), split by dementia and non-dementia cases. The mean, standard deviation (SD), frequencies and percentages were reported. Characteristics of the DSRS total score (e.g., mean, SD, skewness, kurtosis) were reported alongside supplementary characteristics at an item level. The internal consistency of the DSRS was reported to ensure that the items reliably measured the same construct. Macdonald’s omega with 95% Confidence Intervals (CIs) were reported (Hancock & An, 2020). High values represent greater reliability, with a threshold of .7 deemed acceptable internal consistency.

Concurrent validity was checked through a Pearson’s correlation between the DSRS total score against functional and cognitive outcomes, including the CSID relscore, CSID cogscore and the Lawton IADL. A significant correlation (*p* < .05) was used as evidence of concurrent validity.

A multiple regression model was developed to understand the factors associated with the DSRS total score. The model was created in three stages. In the first stage, non-modifiable risk factors for dementia (age and sex) were entered. In the second stage, older adult education attainment was entered, and in the final stage, factors associated with informant characteristics were entered into the model (familial informant, cohabiting informant, informant education attainment).

The DSRS total score was compared between those with and without dementia in an unadjusted and adjusted model. The adjusted model included theoretically driven factors that might influence performance on the DSRS. To better understand the DSRS screening tool, we identified the optimum threshold to screen for dementia. To achieve this, we generated different thresholds for the DSRS and explored the sensitivity, specificity, Areas Under Curve (AUCs) and Youden Index (J) for each. The classification of the AUC Receiver Operating Characteristics (ROC) score was as follows: AUC <.5 was considered no discrimination, 0.6 ≥ AUC > 0.5 was considered poor discrimination, 0.7 ≥ AUC > .6 was considered acceptable discrimination, 0.9 ≥ AUC > .7 was considered excellent discrimination, and AUC > 0.9 was considered outstanding discrimination (Yang & Berdine, 2017). For the Youden Index, higher scores represented better agreement, but no rule of thumb thresholds were applied.

Following assessment of agreement for the overall sample, we then sought to understand the influence of significant covariates (identified in the multiple regression model, outside of age, sex, and older adult education attainment) on AUCs for the optimal threshold. We calculated critical z-ratios to determine whether AUCs differed between subgroups (Hanley & McNeil, 1983). If the z-ratio was above 1.96, it indicated that the AUCs were significantly different and that the covariate might influence the implementation of the screening tool in practice.

A sample size calculation was completed prior to data collection to estimate the sample required to detect with sufficient precision our primary outcome, dementia prevalence. There was an expected dementia prevalence of 4.5% with a precision of ±.9% within each country. As such, we had a target sample size of 2039 participants for each country. A *p*-value <.05 was used to identify statistical significance. All data analysis was performed using IBM SPSS Version 28.

## Results

### Participants

A total of 2110 older adults in Indonesia and 408 older adults in South Africa had sufficient data for inclusion in the study. 562 cases (26.6%) and 59 cases (14.5%) of dementia were identified, respectively. Descriptive data are reported in Table 1.

**Table 1.** Demographic participants in Indonesia (n = 2110) and South Africa (n = 408), split in dementia caseness based on the 10/66 short algorithm.

	Indonesia				South Africa			
Variable	Missing	Dementia (n = 562)	No dementia (n = 1548)	Total (n = 2110)	Missing	Dementia (n = 59)	No dementia (n = 349)	Total (n = 408)
	n	M(SD)	M(SD)	M(SD)	n	M(SD)	M(SD)	M(SD)
Age	0	73.07 (6.32)	70.39 (4.88)	71.10 (5.43)	0	80.88 (7.81)	73.71 (6.81)	74.76 (7.42)
	n	n (%)	n (%)	n (%)	n	n (%)	n (%)	n (%)
Sex: Male	0	177 (31.5%)	676 (43.7%)	853 (40.4%)	16	18 (34.0%)	115 (33.9%)	133 (34.0%)
Older adult education level: Less then primary school	31	286 (51.7%)	500 (32.8%)	786 (37.8%)	14	34 (64.2%)	132 (38.7%)	166 (42.1%)
Informant education level: Less than primary school	1	60 (10.7%)	143 (10.7%)	203 (9.6%)	0	5 (8.5%)	29 (8.3%)	34 (8.3%)
Informant residential status: Cohabiting	6	449 (80.0%)	1208 (78.3%)	1657 (78.8%)	1	81 (86.4%)	269 (78.0%)	320 (79.2%)
Informant relationship: Family member	0	480 (85.4%)	1306 (84.4%)	1786 (84.6%)	4	49 (83.1%)	272 (78.2%)	321 (78.9%)

### Dementia severity rating scale data: Whole sample

Following data imputation there were 2108 cases in Indonesia and 408 cases in South Africa with both DSRS and 10/66 short schedule data. On the DSRS, participants on average scored 3.72 (SD = 6.03) in Indonesian cohort, and 2.91 (SD = 5.03) in the South African cohort. The DSRS was highly negatively skewed in both countries (Indonesia, skewness = 3.12, and kurtosis = 13.49; South Africa, skewness = 3.24, kurtosis = 14.99). The individual item scores are summarised in Appendix A, Supplementary Tables 1 and 2. In Indonesia, interviewers rated that they were confident in data collected in nearly all instances (n = 2,103, 99.7%), and a minority had “a few doubts” (n = 3, .1%). There were four cases with missing data on this outcome (.2%). A similar pattern was observed in South Africa; interviewers felt confident with the data in 407 cases (99.8%), whilst in one case (.2%) the interviewer expressed “a few doubts.”

### Internal consistency

The DSRS demonstrated an excellent internal consistency in Indonesia (ω = .87, 95% CIs = .85–.89) and South Africa (ω = .84, 95% CIs = .78–.89).

### Concurrent validity

The DSRS was significantly correlated with measures of cognitive and functional impairment in Indonesia and South Africa (*p* < .001). The CSID relscore (Indonesia, r = .64, 95% CIs = .62–.67; South Africa, r = .65, 95% CIs = .59–.70), CSID cogscore (Indonesia, r = −.47, 95% CIs = −.50 to −.43; South Africa, r = −.53, 95% CIs = −.60 to −.46) and Lawton IADL (Indonesia, r = −.54, 95% CIs −.57 to −.51; South Africa, r = −.74, 95% −.77 to −.69).

### Factors associated with dementia severity rating scale score

Multiple regression modelling revealed that higher DSRS scores were associated with increased age in both countries. In addition, within the Indonesian sample, being male, cohabiting informants, and low informant education attainment were also associated with higher DSRS scores. Low older adult education attainment was associated with higher DSRS scores in South Africa but failed to reach statistical significance (*p* = .06) in Indonesia. Having a family informant was not associated with DSRS scores in either model. See Table 2 for further information. Both models had a good fit (*p* < .05), but only accounted for 5% of the variance in the Indonesian sample, and 9% of variance in the South African sample.

**Table 2.** Multiple regression models with DSRS total score as the dependent variable.

Model	Unstandardized coefficients		Standardized coefficients	*p*-value	95.0% Confidence interval for B	
	B	Std. Error	Beta		Lower bound	Upper bound
Indonesia						
(Constant)	−8.90	1.84		<0.001	−12.51	−5.30
Age	0.19	0.02	0.17	<0.001	0.14	0.23
Sex: Male	−1.24	0.28	−0.10	<0.001	−1.79	−0.70
Education attainment: Less than primary	0.53	0.28	0.04	0.06	−0.02	1.08
Informant relationship: Family member	0.18	0.38	0.01	0.63	−0.56	0.93
Informant residential status: Cohabiting	0.94	0.33	0.06	<0.01	0.29	1.59
Informant education attainment: Less than primary	1.19	0.46	0.06	−0.01	0.29	2.09
South Africa						
(Constant)	−11.07	2.54		<0.001	−16.06	−6.08
Age	0.18	0.03	0.28	<0.001	0.12	0.24
Sex: Male	0.04	0.49	0.00	0.94	−0.93	1.01
Education attainment: Less than primary	1.10	0.48	0.12	0.02	0.16	2.04
Informant relationship: Family member	0.41	0.61	0.03	0.50	−0.79	1.60
Informant residential status: Cohabiting	−0.51	0.61	−0.04	0.40	−1.70	0.69
Informant education attainment: Less than primary	−0.33	0.85	−0.02	0.70	−2.01	1.35

### Dementia severity rating scale characteristics: Dementia versus non-dementia

Overall, the DSRS scores were significantly higher in those with dementia, compared to those without dementia, as identified on the 10/66 short schedule (*p* < 0.05). The difference between groups remained significant after controlling for key factors: older adult age, older adult sex, older adult education attainment, familial informant, informant education attainment and cohabiting informant (*p* < .05). See Table 3.

**Table 3.** DSRS characteristics of the participants with and without dementia based on the 10/66 short algorithm. Regression co-efficients (B) alongside robust standard errors are reported for differences between groups.

	Dementia	No dementia	Unadjusted	Adjusted^a^
	M (SD)	M (SD)	B (95% CIs)	B (95% CIs)
Indonesia	8.88 (8.83)	1.84 (2.85)	7.04 (6.30–7.78)	6.87 (6.12–7.62)
South Africa	10.05 (8.17)	1.70 (2.90)	8.35 (6.22–10.48)	7.20 (5.04–9.36)

^a^Adjusted for age (older adult), sex (older adult), low education attainment (older adult and informant), familial informant, cohabiting informant.

Note, two cases were missing data sufficient to calculate the DSRS score.

### The dementia severity rating scale as a screening tool

A score >2 on the DSRS revealed to have the greatest agreement with the 10/66 short algorithm based on the Youdon Index in Indonesia (J = .49) and South Africa (J = .64). Applying this threshold had excellent discrimination properties in Indonesia (AUC = .75, 95% CIs = .72–.77) and South Africa (AUC = .82, 95% CIs = .76–.88). See Table 4 for the sensitivity, specificity and Youden index for each threshold. Applying the threshold DSRS >2, the DSRS demonstrated excellent agreement with the 10/66 short diagnostic schedule for non-cohabiting informants and cohabiting informants in both countries. There was no significant difference between the AUCs for cohabiting and non-cohabiting informants in Indonesia and South Africa (*p* > .05). Low and high informant education attainment groups demonstrated excellent agreement in both countries. There was no significant difference between the AUCs for low and high education attainment in Indonesia and South Africa (*p* > .05). See Table 5 for further details.

**Table 4.** The sensitivity, specificity and Youden index after applying different thresholds to the DSRS total score in comparison to the 10/66 short algorithm.

DSRS threshold	Indonesia			South Africa		
	Sensitivity	Specificity	Youden	Sensitivity	Specificity	Youden
>0	0.902	0.474	0.376	0.949	0.513	0.462
>1	0.840	0.640	0.480	0.898	0.711	0.609
>2	**0.747**	**0.747**	**0.494**	**0.864**	**0.771**	**0.635**
>3	0.676	0.817	0.493	0.797	0.819	0.616
>4	0.616	0.862	0.477	0.763	0.868	0.631
>5	0.557	0.904	0.461	0.712	0.908	0.620
>6	0.489	0.925	0.414	0.678	0.923	0.601
>7	0.441	0.942	0.383	0.593	0.940	0.533
>8	0.381	0.955	0.336	0.508	0.954	0.463

Note: Further thresholds were explored but performed poorer (based on the Youden Index) than those reported. Bold font represents optimum DSRS threshold based on highest Youden Index within each country.

**Table 5.** The AUCs for the DSRS threshold (>2) to identify cases of dementia against the 10/66 short algorithm, split by residential status and informant education attainment subgroups. Z-ratios are reported to describe the difference between groups.

	Indonesia		South Africa	
	AUC (95%CIs)	z	AUC (95%CIs)	z
Residential status		0.78		−0.42
Co-habiting	0.74 (0.72–0.77)		0.82 (0.76–0.89)	
Non co-habiting	0.76 (0.70–0.81)		0.79 (0.64–0.94)	
Informant education		−0.29		0.37
Low education	0.76 (0.68–0.83)		0.78 (0.55–1.00)	
High education	0.75 (0.72–0.77)		0.82 (0.76–0.88)	

## Discussion

This is the first study to explore the properties of the DSRS and its potential for it to be adopted as a dementia screening tool in LMICs. The findings indicate that the DSRS demonstrates good psychometric properties, and that in adopting a threshold of >2 is excellent in distinguishing between dementia and non-dementia cases. These data suggest that the DSRS appears to have good properties within both countries, with very few missing data points, and interviewers rated that they were confident with the data collected in nearly all instances (>99%). Our findings show that DSRS demonstrated excellent internal consistency (ω = .84–.87), in line with previous research (α = .92) (Clark & Ewbank, 1996). It is reassuring that the DSRS had good concurrent validity against measures of cognitive and functional impairment.

DSRS scores were found to be associated with factors such as age, sex and older adult education attainment, albeit not consistently across the countries. These associations are perhaps not surprising as they all reflect risk factors for dementia (Letenneur et al., 1999). Cohabiting informants were associated with an increased DSRS score in Indonesia, in line with evidence that cohabiting informants are more likely to report greater functional impairment (Hackett et al., 2020). We may theorise that cohabiting informants who spend more time with the older adult are more likely to observe everyday impairment, thus providing more accurate reports (Ready et al., 2004). However, this interpretation may not be the full story since the DSRS has also been shown to have good reliability between caregivers and experienced doctors or non-physician researchers (Clark & Ewbank, 1996). An alternative explanation for the association could be that those with greater impairment are more likely to live with someone who would provide care and support. This might explain why no association was reported between familial informants and the DSRS across both countries. Another interesting finding was that lower informant education attainment was associated with higher scores on the DSRS within the Indonesian cohort. Previous research has identified that informant education levels are associated with informant reports of functional impairment (Hackett et al., 2020), perhaps indicating issues with comprehension. No such association was reported in the South African sample, potentially attributable to the smaller sample size.

Our findings identify that DSRS scores significantly differed between dementia and non-dementia cases and remained robust even after controlling for covariates. Following an explorative stage, we identified that by applying a threshold of >2 to identify cases of dementia, the DSRS has in excellent agreement with the 10/66 short diagnostic algorithm in both countries. This same threshold has been used to distinguish between AD and healthy controls (Roalf et al., 2013), and while in our LMIC population the properties were lower compared to those reported by Roalf and colleagues (AUC = .99), it further strengthens our findings. Neither cohabiting informant status nor informant education attainment significantly influenced the accuracy sufficient to change the overall AUCs based on a priori interpretations (i.e., excellent agreement was observed irrespective of subgroup).

Our study has several important limitations. First, while informants were selected on the basis that they had sufficient insight into the older adult’s life, we did not have an index of this. The use of measures describing whether the informant was familial or cohabiting are likely to be good proxies of this but are not a guarantee. Second, we did not explore whether the DSRS should be scored differently, as at present, all domains are equally weighted, albeit with varying maximum scores. Third, our results are predicated on the 10/66 short diagnostic algorithm being accurate. There is growing evidence of its validity (Abdin et al., 2017; Ibnidris et al., 2021; Khan & Prince, 2022; Stewart et al., 2016), but elevated prevalence rates in some populations, like older people with low education, raise some concerns. Fourth, our findings reflect those from only two middle-income countries, thus further research is needed within other settings prior to adoption. Fifth, whilst we did not employ an a priori sample size calculation for the analysis reported here, post hoc analysis reveals adequate AUC precision for the Indonesian data (CI width = .05, Confidence Level = .95). Unfortunately, due to difficulties recruiting because of the COVID-19 pandemic, we failed to reach our target sample size in South Africa, thus reducing AUC precision (CI width = .12, Confidence Level = .95). Whilst further research with a larger sample size is recommended to improve precision of estimates, our present CIs for AUCs do not cross previous definitions of agreement (i.e., .9 ≥ AUC> .7 was considered excellent discrimination) at a whole sample level. Caution should, however, be taken when interpreting subgroup analysis, particularly within the South African sample. Finally, the DSRS is reliant on there being someone with sufficient insight into the older adult’s life. It cannot therefore be used in those who are truly isolated.

## Conclusion

The DSRS is a short instrument, which is cost free, and does not require special examination skills to complete. Our study highlights that within two middle-income countries it demonstrates excellent psychometric properties and accuracy in identifying cases of dementia when compared against a standardised diagnostic algorithm. Although the DSRS appears to be somewhat sensitive to informant education attainment and cohabiting informant status, the mechanism of association is unclear and does not undermine its screening ability. Further research is needed to validate it further against clinical diagnostic criteria, and against other informant screening tools. However, informant tools such as the DSRS may have an important role in dementia screening in low and middle-income healthcare settings.

## Supplemental Material

Supplemental Material - The dementia severity rating scale: A potential community screening tool for dementia in low- and middle-income countries Supplemental Material for The dementia severity rating scale: A potential community screening tool for dementia in low- and middle-income countries by Yuda Turana, Nicolas Farina, Imelda Theresia, Fasihah Irfani Fitri, Ika Suswanti, Roxanne Jacobs, Marguerite Schneider, Tara Puspitarini Sani, Adelina Comas-Herrera, Emiliano Albanese, Ishtar Govia, Cleusa P Ferri, Martin Knapp and Sube Ban in Dementia

## Data Availability

Data will become available on UK Data Services by 2024 and is available from the corresponding author, N.F.*

## References

[bibr1-14713012231186837] Abdin E. Vaingankar J. A. Picco L. Chua B. Y. Prince M. Chong S. A. Subramaniam M. (2017). Validation of the short version of the 10/66 dementia diagnosis in multiethnic Asian older adults in Singapore. BMC Geriatrics, 17(1), 94. 10.1186/s12877-017-0475-7. 28431511 PMC5399400

[bibr2-14713012231186837] Baldo J. V. Arévalo A. Patterson J. P. Dronkers N. F. (2013). Grey and white matter correlates of picture naming: Evidence from a voxel-based lesion analysis of the Boston Naming Test. Cortex; a Journal Devoted to the Study of the Nervous System and Behavior, 49(3), 658–667. 10.1016/j.cortex.2012.03.001. 22482693 PMC3613759

[bibr3-14713012231186837] BPS-Statistics Indonesia . (2019). Statistik Indonesia: Statistical yearbook of Indonesia 2019. BPS-Statistics Indonesia.

[bibr4-14713012231186837] Burns R. Nichols L. O. Graney M. J. Martindale-Adams J. Lummus A. (2006). Cognitive abilities of Alzheimer’s patients: Perceptions of black and white caregivers. International Journal of Aging & Human Development, 62(3), 209–219. 10.2190/3GG6-8YV1-ECJG-8XWN. 16625937

[bibr5-14713012231186837] Clark C. M. Ewbank D. C. (1996). Performance of the dementia severity rating scale: A caregiver questionnaire for rating severity in Alzheimer disease. Alzheimer Disease & Associated Disorders, 10(1), 31–39. 10.1097/00002093-199601010-00006. 8919494

[bibr6-14713012231186837] Creavin S. T. Wisniewski S. Noel-Storr A. H. Trevelyan C. M. Hampton T. Rayment D. Thom V. M. Nash K. J. E. Elhamoui H. Milligan R. Patel A. S. Tsivos D. V. Wing T. Phillips E. Kellman S. M. Hannah L. Shackleton G. F. S. Neale B. E. Watton M. E. Cullu S. (2016). Mini-Mental State Examination (MMSE) for the detection of dementia in clinically unevaluated people aged 65 and over in community and primary care populations (Review). Cochrane Database of Systematic Reviews, 1(4), 1–23. 10.1002/14651858.CD011145.pub2. www.cochranelibrary.com PMC8812342 26760674

[bibr7-14713012231186837] Farina N. Jacobs R. Sani T. P. Schneider M. Theresia I. Turana Y. Fitri F. I. Albanese E. Lorenz-Dant K. Docrat S. Toit P. D. Ferri C. P. Govia I. Comas-Herrera A. Ibnidris A. Knapp M. Banerjee S. (2022). Description of the cross-cultural process adopted in the STRiDE (STrengthening responses to dementia in DEveloping countries) program: A methodological overview. Alzheimer’s & dementia (Amsterdam, Netherlands), 14(1), Article e12293. 10.1002/dad2.12293. PMC8923343 35317433

[bibr8-14713012231186837] Farina N. Jacobs R. Turana Y. Fitri F. I. Schneider M. Theresia I. Docrat S. Sani T. P. Augustina L. Albanese E. Comas-Herrera A. Du Toit P. Ferri C. P. Govia I. Ibnidris A. Knapp M. Banerjee S. (2023). Comprehensive measurement of the prevalence of dementia in low- and middle-income countries: STRiDE methodology and its application in Indonesia and South Africa. BJPsych Open, 9(4), Article e102. 10.1192/bjo.2023.76. 37278200 PMC10305093

[bibr9-14713012231186837] Gauthier S. Rosa-Neto P. Morais J. A. Webster C. (2021). World Alzheimer Report 2021: Journey through the diagnosis of dementia. London, UK: Alzheimer's Disease International. 17–29.

[bibr10-14713012231186837] Nichols E. Steinmetz J. D. Vollset S. E. Fukutaki K. Chalek J. Abd-Allah F. Abdoli A. Abualhasan A. Abu-Gharbieh E. Akram T. T. Al Hamad H. Alahdab F. Alanezi F. M. Alipour V. Almustanyir S. Amu H. Ansari I. Arabloo J. Ashraf T. Vos T. GBD 2019 Dementia Forecasting Collaborators . (2022). Estimation of the global prevalence of dementia in 2019 and forecasted prevalence in 2050: An analysis for the Global Burden of Disease Study 2019. The Lancet. Public health, 7(2), e105–e125. 10.1016/S2468-2667(21)00249-8. 34998485 PMC8810394

[bibr11-14713012231186837] Hackett K. Mis R. Drabick D. A. G. Giovannetti T. (2020). Informant reporting in mild cognitive impairment: Sources of discrepancy on the functional activities questionnaire. Journal of the International Neuropsychological Society: JINS, 26(5), 503–514. 10.1017/S1355617719001449. 31964443 PMC7205566

[bibr12-14713012231186837] Hall K. S. Gao S. Emsley C. L. Ogunniyi A. O. Morgan O. Hendrie H. C. (2000). Community screening interview for dementia (CSI ‘D’); performance in five disparate study sites. International journal of geriatric psychiatry, 15(6), 521–531. 10.1002/1099-1166(200006)15:6<521::aid-gps182>3.0.co;2-f. 10861918

[bibr13-14713012231186837] Hancock G. R. An J. (2020). A closed-form alternative for estimating ω reliability under unidimensionality. Measurement: Interdisciplinary Research and Perspectives, 18(1), 1–14. 10.1080/15366367.2019.1656049.

[bibr14-14713012231186837] Hanley J. A. McNeil B. J. (1983). A method of comparing the areas under receiver operating characteristic curves derived from the same cases. Radiology, 148(3), 839–843. 10.1148/radiology.148.3.6878708. 6878708

[bibr15-14713012231186837] Harris P. A. Taylor R. Minor B. L. Elliott V. Fernandez M. O'Neal L. Mcleod L. Delacqua G. Delacqua F. Kirby J. Duda S. N. REDCap Consortium . (2019). The REDCap consortium: Building an international community of software platform partners. Journal of Biomedical Informatics, 95(103208), 103208. 10.1016/j.jbi.2019.103208. 31078660 PMC7254481

[bibr16-14713012231186837] Harris P. A. Taylor R. Thielke R. Payne J. Gonzalez N. Conde J. G. (2009). Research electronic data capture (REDCap)-A metadata-driven methodology and workflow process for providing translational research informatics support. Journal of Biomedical Informatics, 42(2), 377–381. 10.1016/j.jbi.2008.08.010. 18929686 PMC2700030

[bibr17-14713012231186837] Ibnidris A. Piumatti G. Carlevaro F. Fadda M. Magno F. Magistro D. Albanese E. (2021). Italian version of the short 10/66 dementia diagnostic schedule: A validation study. BMJ Open, 11(6), Article e045867. 10.1136/bmjopen-2020-045867. PMC8246379 34193490

[bibr18-14713012231186837] Jorm A. F. (2003). The value of informant reports for assessment and prediction of dementia. Journal of the American Geriatrics Society, 51(6), 881–882. 10.1046/j.1365-2389.2003.51276.x. 12757582

[bibr19-14713012231186837] Khan Q. U. A. Prince M. J. (2022). Validation of the short version of the 10/66 dementia diagnosis in Urdu in Karachi, Pakistan. Alzheimer disease and associated disorders, 36(1), 89–91. 10.1097/WAD.0000000000000467. 34310442

[bibr20-14713012231186837] Khuluvhe M. (2021). Adult illiteracy in South Africa. Higher Education & Training, March.

[bibr21-14713012231186837] Larner A. J. (2012). Screening utility of the montreal cognitive assessment (MoCA): In place of—or as well as—the MMSE? International Psychogeriatrics, 24(3), 391–396. 10.1017/S1041610211001839. 22014176

[bibr22-14713012231186837] Lawton M. P. Brody E. M. (1969). Assessment of older people: Self-maintaining and instrumental activities of daily living. The Gerontologist, 9(3 Part 1), 179–186. 10.1093/geront/9.3_Part_1.179. 5349366

[bibr23-14713012231186837] Letenneur L. Gilleron V. Commenges D. Helmer C. Orgogozo J. M. Dartigues J. F. (1999). Are sex and educational level independent predictors of dementia and Alzheimer’s disease? Incidence data from the PAQUID project. Journal of Neurology Neurosurgery and Psychiatry, 66(2), 177–183. 10.1136/jnnp.66.2.177. 10071096 PMC1736218

[bibr24-14713012231186837] Li F. Jia X. F. Jia J. (2012). The informant questionnaire on cognitive decline in the elderly individuals in screening mild cognitive impairment with or without functional impairment. Journal of Geriatric Psychiatry and Neurology, 25(4), 227–232. 10.1177/0891988712464822. 23172761

[bibr26-14713012231186837] McKhann G. M. Knopman D. S. Chertkow H. Hyman B. T. Jack C. R. Jr. Kawas C. H. Klunk W. E. Koroshetz W. J. Manly J. J. Mayeux R. Mohs R. C. Morris J. C. Rossor M. N. Scheltens P. Carrillo M. C. Thies B. Weintraub S. Phelpsw C. H. Phelps C. H. (2011). The diagnosis of dementia due to Alzheimer’s disease: Recommendations from the National Institute on Aging Alzheimer’s Association workgroups on diagnostic guidelines for Alzheimer’s disease. Alzheimer's & dementia: the journal of the Alzheimer's Association, 7(3), 263–269. 10.1016/j.jalz.2011.03.005. PMC3312024 21514250

[bibr27-14713012231186837] Morris J. C. Heyman A. Mohs R. C. Hughes J. P. van Belle G. Fillenbaum G. Mellits E. D. Clark C. (1989). The consortium to establish a Registry for Alzheimer's disease (CERAD). Part I. Clinical and neuropsychological assessment of Alzheimer's disease. Neurology, 39(9), 1159–1165. 10.1212/wnl.39.9.1159. 2771064

[bibr28-14713012231186837] Nasreddine Z. S. Phillips N. A. Bédirian V. Charbonneau S. Whitehead V. Collin I. Cummings J. L. Chertkow H. (2005). The montreal cognitive assessment, MoCA: A brief screening tool for mild cognitive impairment. Journal of the American Geriatrics Society, 53(4), 695–699. 10.1111/j.1532-5415.2005.53221.x. 15817019

[bibr29-14713012231186837] Perroco T. R. Bustamante S. E. Z. Moreno M. D. P. Q. Hototian S. R. Lopes M. A. Azevedo D. Litvoc J. Filho W. J. Bottino C. M. C. (2009). Performance of Brazilian long and short IQCODE on the screening of dementia in elderly people with low education. International Psychogeriatrics, 21(3), 531–538. 10.1017/S1041610209008849. 19323868

[bibr30-14713012231186837] Prince M. Bryce R. Albanese E. Wimo A. Ribeiro W. Ferri C. P. (2013). The global prevalence of dementia: A systematic review and metaanalysis. Alzheimer's & dementia: the journal of the Alzheimer's Association, 9(1), 63–75.e2. 10.1016/j.jalz.2012.11.007. 23305823

[bibr31-14713012231186837] Prince M. J. Wimo A. Guerchet M. M. Ali G.-C. Wu Y.-T. Prina M . (2015). World Alzheimer Report 2015: The Global Impact of Dementia: An analysis of prevalence, incidence, cost and trends. London, UK: Alzheimer’s Disease International.

[bibr32-14713012231186837] Prince M. J. Beekman A. T. F. Deeg D. J. H. Fuhrer R. Kivela S. L. Lawlor B. A. Lobo A. Magnusson H. Meller I. Van Oyen H. Reischies F. Roelands M. Skoog I. Turrina C. Copeland J. R. M. (1999). Depression symptoms in late life assessed using the EURO-D scale. Effect of age, gender and marital status in 14 European centres. The British journal of psychiatry: the journal of mental science, 174(4), 339–345. 10.1192/bjp.174.4.339. 10533553

[bibr33-14713012231186837] Ready R. E. Ott B. R. Grace J. (2004). Validity of informant reports about AD and MCI patients’ memory. Alzheimer Disease and Associated Disorders, 18(1), 11–16. 10.1097/00002093-200401000-00003. 15195458

[bibr34-14713012231186837] Roalf D. R. Moberg P. J. Xie S. X. Wolk D. A. Moelter S. T. Arnold S. E. (2013). Comparative accuracies of two common screening instruments for classification of Alzheimer's disease, mild cognitive impairment, and healthy aging. Alzheimer’s & dementia: the journal of the Alzheimer’s Association, 9(5), 529–537. 10.1016/j.jalz.2012.10.001. PMC4036230 23260866

[bibr35-14713012231186837] Stewart R. Guerchet M. Prince M. (2016). Development of a brief assessment and algorithm for ascertaining dementia in low-income and middle-income countries: The 10/66 short dementia diagnostic schedule. BMJ Open, 6(5), Article e010712. 10.1136/bmjopen-2015-010712. PMC4885443 27225649

[bibr36-14713012231186837] STRiDE Project (2018). Strengthening responses to dementia in developing countries (STRiDE). https://stride-dementia.org/about-the-project/

[bibr37-14713012231186837] Taylor-Rowan M. Nafisi S. Owen R. Duffy R. Patel A. Burton J. K. Quinn T. J. (2023). Informant-based screening tools for dementia: An overview of systematic reviews. Psychological Medicine, 53(2), 580–589. 10.1017/S0033291721002002. 34030753

[bibr38-14713012231186837] Turana Y. Handajani Y. S. (2011). Mini-Mental State Examination (MMSE) normative value: Based on age and level of education In Elderly in Jakarta. Medika, Tahun ke X, 307–310.

[bibr40-14713012231186837] Wang X. Li F. Gao Q. Jiang Z. Abudusaimaiti X. Yao J. Zhu H . (2022). Evaluation of the Accuracy of Cognitive Screening Tests in Detecting Dementia Associated with Alzheimer’s Disease: A Hierarchical Bayesian Latent Class Meta-Analysis. Journal of Alzheimer’s Disease, 87(1), 285–304. 10.3233/JAD-215394. 35275533

[bibr39-14713012231186837] Yang S. Berdine G. (2017). The receiver operating characteristic (ROC) curve. The Southwest Respiratory and Critical Care Chronicles, 5(19), 34. 10.12746/swrccc.v5i19.391.

