# Supplementary material for: The dementia severity rating scale: A potential community screening tool for dementia in low- and middle-income countries
Source: Dementia (London). 2023 Dec 14;23(3):476–92. doi: 10.1177/14713012231186837 (PMC11041075; doi:10.1177/14713012231186837)
Supplement: Supplemental Material - The dementia severity rating scale: A potential community screening tool for dementia in low- and middle-income countries [file sj-pdf-1-dem-10.1177_14713012231186837.pdf]

Appendix A

Indonesia

- Every item of the DSRS had at least two missing data points. The item with the least number of missing data were related to home-based activities (n=2, 0.1%). The item with the most missing data were related to orientation to place (n=14, 0.7%).
- There were 51 cases (2.4%) of missing data on the DSRS, in which 45 cases were due to one missing item, four cases were due to two missing items, and two cases in which no data were collected.
- The mode item value was imputed in 53 data points across 49 cases.
- Without data imputation (n=2,059), on average participants score 3.74 (SD=6.06), and ranged of 51 (min=0, max=51). The DSRS was highly negatively skewed, skewness = 3.12, kurtosis = 13.49.

Supplementary Table 1. Individual item characteristics of the DSRS (n=2,108), of which 49 cases included imputation.

	Mean	SD	Median	Percentage Normal, n (Valid %)	Skewness	Kurtosis
Memory	0.72	0.96	0	1089 (51.7%)	1.78	4.19
Speech	0.26	0.71	0	1729 (82.0%)	4.11	21.56
Recognition	0.15	0.51	0	1892 (89.8%)	4.09	19.12
Orientation to time	0.36	0.68	0	1514 (71.8%)	2.46	7.65
Orientation to place	0.21	0.59	0	1798 (85.3%)	3.71	16.10
Decision making	0.34	0.8	0	1667 (79.1%)	2.82	8.07
Social activity	0.32	0.94	0	1785 (84.7%)	3.27	10.02
Home	0.58	1.21	0	1557 (73.9%)	2.15	3.20
Personal care	0.14	0.53	0	1956 (92.8%)	4.13	16.63
Eating	0.09	0.41	0	1992 (94.5%)	5.37	30.68
Toilet	0.13	0.6	0	1983 (94.1%)	4.98	25.20

Transport	0.41	1.11	0	1778 (84.3%)	3.04	9.20

Note. Of the 49 imputed cases, 53 data points were imputed. The data exclude two cases in which imputation was not feasible due to missing all items of the DSRS.

South Africa

- Memory, recognition, orientation to time, orientation to place, social activities, and home-based activities had no missing data points. The item with the most missing data were related to language, eating, and travel (n=3, 0.7%).
- There were 12 cases (2.9%) of missing data on the DSRS, in which 11 cases were due to one missing item, and one case in which there were two missing items.
- The mode item value was imputed in 13 data points across 12 cases.
- Without data imputation (n=396), on average participants score 2.92 (SD=5.07), and ranged of 39 (min=0, max=38). The DSRS was highly negatively skewed, skewness = 3.26, kurtosis = 15.05.

Supplementary Table 2. Individual item characteristics of the DSRS (n=408), following imputation (n=12).

	Mean	SD	Median	IQR	Percentage Normal, n (Valid %)	Skewness	Kurtosis
Memory	0.45	0.72	0.00	1	266 (65.2%)	2.06	6.01
Speech	0.10	0.49	0.00	0	382 (93.6%)	6.24	43.51
Recognition	0.12	0.54	0.00	0	381 (93.4%)	5.73	38.86
Orientation to time	0.18	0.55	0.00	0	356 (87.3%)	4.18	21.25
Orientation to place	0.07	0.44	0.00	0	391 (95.8%)	7.64	63.41
Decision making	0.23	0.63	0.00	0	344 (84.3%)	3.25	11.66
Social activity	0.75	1.39	0.00	1	294 (72.1%)	1.67	1.23
Home	0.48	1.04	0.00	1	305 (74.8%)	2.43	4.98
Personal care	0.10	0.43	0.00	0	384 (94.1%)	4.93	25.26
Eating	0.03	0.20	0.00	0	400 (98.0%)	8.21	70.92
Toilet	0.11	0.51	0.00	0	385 (94.4%)	5.64	33.86
Transport	0.29	0.91	0.00	0	356 (87.3%)	3.90	16.48

Note. Of the 12 imputed cases, there were 13 data points imputed.
